# Sensitivity of renal cell carcinoma to cuproptosis: a bioinformatics analysis and experimental verification

**DOI:** 10.7150/jca.103708

**Published:** 2025-01-01

**Authors:** Hongfang Li, Chanjuan Zhang, Neng Zhu, Yaning Shi, Li Qin

**Affiliations:** 1 Laboratory of Stem Cell Regulation with Chinese Medicine and Its Application, School of Pharmacy, Hunan University of Chinese Medicine, Changsha 410208, China; 2 Department of Urology, The First Hospital of Hunan University of Chinese Medicine, Changsha 410007, China; 3 Institutional Key Laboratory of Vascular Biology and Translational Medicine in Hunan Province, Changsha, China; 4 Hunan Province Engineering Research Center of Bioactive Substance Discovery of Traditional Chinese Medicine, Hunan University of Chinese Medicine, Changsha, China.

**Keywords:** Cuproptosis, Clear cell renal cell carcinoma, FDX1, DLAT

## Abstract

**Purpose:** Targeting cuproptosis is considered as a promising therapeutic strategy for the prevention of tumors. However, the potential role of cuproptosis and its related genes in clear cell renal cell carcinoma (ccRCC) remains elusive. The present study aims to explore the sensitivity of ccRCC to cuproptosis and its underlying mechanism.

**Methods:** Cuproptosis differential genes (CDGs) were extracted using the GSE53757 and GSE66272 datasets. A comprehensive analysis of the role of CDGs was conducted through multiple public databases and experiments.

**Results:** It was found that cuproptosis inducer elesclomol significantly induced cell death in 786-O and A498 cells. FDX and DLAT exhibited significantly low expression, which were independent prognostic factors for poor survival, and had a strong positive correlation in ccRCC patients. Functional analysis of differentially expressed genes positively or negatively correlated with both FDX1 and DLAT indicated that acetyl-CoA biosynthetic process and acetyl-CoA metabolic process were remarkably affected. In ccRCC patients, the methylation levels and sites of FDX1 and DLAT genes were dramatically correlated with overall survival (OS). The expressions of FDX1 and DLAT were closely related to immune infiltration and immune checkpoints. Docking results indicated that mitotane, adicicol and dihydrolipoic acid might be potential drug targets for FDX1 and DLAT.

**Conclusions:** Overall, the present study demonstrates the sensitivity of ccRCC to cuproptosis, and targeting the combination of FDX1 and DLAT may be a novel therapeutic strategy to induce cuproptosis in ccRCC.

## Introduction

Clear cell renal cell carcinoma (ccRCC), a major histological subtype of RCCs, accounts for 70%-80% of all renal cell carcinoma cases^1^, ^2^. At present, early-stage ccRCC can usually be treated effectively by surgery, but the treatment of advanced ccRCC still faces severe clinical challenges^3^. Although early detection and timely treatment are beneficial for ccRCC patients, clinically successful ccRCC-specific markers are still lacking^4^, ^5^. As such, there is an urgent require to identify sensitive molecular biomarkers and therapeutic strategies for ccRCC therapy.

Programmed cell death (PCD) is a type of gene-regulated cell death that not only plays an important role in fundamental biological processes but also participates in a variety of pathological processes^6^. An increasing number of PCD types, such as apoptosis, necroptosis, autophagy, ferroptosis and pyroptosis, have been implicated in the pathological and physiological processes of various diseases, especially tumors^7^, ^8^. A typical example is that targeting autophagy to treat tumors has brought enormous clinical benefits to cancer patients^9^, ^10^. For example, gefitinib, lapatinib and erlotinib are common clinical tyrosine kinase inhibitors that target autophagy to treat breast cancer, chronic myeloid leukemia, and gastrointestinal stromal tumors^11^, ^12^. However, resistance to autophagy is largely responsible for the failure of these therapeutic strategies^13^. Different forms of PCD can be substituted for each other in the treatment of tumors^14^. Therefore, finding novel forms of PCD is crucial for therapy-resistant tumors. More recently, a novel regulated form of cell death, termed cuproptosis, has attracted considerable academic interest, referring to copper ionophore-induced (including elesclomol, disulfiram and NSC319726) binding of copper to the lipoylated tricarboxylic acid (TCA) cycle proteins, ultimately stimulating cell death^15^. The progression of ccRCC is closely related to the reprogramming of the TCA cycle based on metabolomics analysis^16^, which regulates energy metabolism through the TCA cycle, allowing tumor cells to survive under nutrient-depleted conditions and escape the immune system^17^, ^18^. Although a previous study has analyzed the prognostic role of cuproptosis-related genes in ccRCC^19^, these theories lacked experimental verification. In particular, the sensitivity of ccRCC to cuproptosis still lacks a large amount of reliable experimental data, and the relationship between cuproptosis related genes and methylation and immune microenvironment remains unclear.

Herein, we sought to find the potential functions of the most critical cuproptosis-related genes in ccRCC on the basis of public databases. Additionally, the protein expression of cuproptosis-related genes was verified in our collected ccRCC clinical samples. Furthermore, the sensitivity of ccRCC to cuproptosis was examined and its potential target genes were evaluated *in vitro* experiments.

## Methods and Materials

### Cuproptosis-related genes and ccRCC differential genes collection and preprocessing

The 10 cuproptosis-related genes were obtained from a previously study^15^. The GSE53757 and GSE66272 datasets are the commonly utilized expression profiles for analyzing differentially expressed genes in ccRCC. Compared with other ccRCC datasets, GSE53757 and GSE66272 contain a relatively large number of samples. Thus, we chose the GSE53757 and GSE66272 cohorts for the differentially analysis. The GSE53757 and GSE66272 datasets was downloaded from the GEO database (https://www.ncbi.nlm.nih.gov/geo/), which includestumor samples and matched adjacent paracancerous tissues from 99 patients with ccRCC. A total of 99 ccRCC tumor tissue and adjunct nontumor samples were analyzed by GEO2R, an online analysis tool, to acquire the differential expression of genes. The differentially expressed genes were selected under the following criteria: |log2 (fold-change) | > 0.5 and *P* < 0.05. Subsequently, cuproptosis-related genes and ccRCC differential genes were intersected to identify CDGs. A venn diagram of differentially expressed genes was created using the online tool Venny2.1.0, and the heatmap and volcano plot of differential expression genes about the gene expression profiles in the GSE53757 and GSE66272 databases were drawn by R software (version 4.0.3; R Foundation).

### The expression, mapping and prognosis of cuproptosis differential genes

Following that, the mRNA expression profile data of CDGs in tumor tissues and different clinical stages of ccRCC, such as pathologic state and OS events, were analyzed using RNA-seq gene expression data and clinical information downloaded from the TCGA database. The protein expression and localization of CDGs were explored by the Human Protein Atlas (HPA, https://www.proteinatlas.org/). The R software packages "Survival" and "Survminer" perform the Kaplan-Meier (KM) survival curve analysis. The univariate Cox regression analysis was used to calculate the relationship between CDGs expression and the ccRCC patient's OS. A multivariate analysis was done to determine whether the CDGs were independent prognostic factors for ccRCC patient survival, and their diagnostic values were assessed with a receiver operating characteristic (ROC) curve analysis.

### Co-expression network and functional enrichment analysis of CDGs

The genes co-expressed with FDX and DLAT in ccRCC were acquired from LinkedOmics (http://linkedomics.org/login.php), and screened according to the Pearson correlation coefficient (|cor| > 0.3, P < 0.05). Heatmaps of the top 50 negative or positive FDX and DLAT expression correlated genes were plotted by the “pheatmap” R package. To demonstrate the biological function of CDGs and the top 50 genes most positively or negatively correlated with FDX and DLAT, gene ontology (GO) enrichment analysis and Kyoto Encyclopedia of Genes and Genomes (KEGG) pathway analysis were performed on the Metascape website (http://metascape.org/gp/index).

### Gene mutation and methylation of ferredoxin 1 (FDX1) and dihydrolipoyl transacetylase (DLAT)

The FDX1 and DLAT gene mutations in ccRCC were evaluated using cBioPortal (http://www.cbioportal.org/). To further investigate the effect of DNA methylation status of FDX1 and DLAT on ccRCC, MEXPRESS visualized expression, DNA methylation, and clinical parameters according to TCGA data^20^. Then, the relative DNA methylation site data for FDX1 and DLAT was evaluated using an online tool called MethSurv (https://biit.cs.ut.ee/methsurv/). In addition, the survival analysis and prognostic value of all methylation sites were explored.

### Analysis of immune infiltration and immune checkpoints

The molecular characterization of tumor immune interactions in ccRCC was investigated using the single-sample GSEA (ssGSEA) approach from the “GSVA” R package and the Tumor Immune Estimation Resource (TIMER) database (http://timer.cistrome.org/). Furthermore, we explored the effect of low or high FDX and DLAT expression on immune cell infiltration. Immune checkpoints were identified using the TISIDB database (http://cis.hku.hk/TISIDB/).

### Drug targets screening and validation

The candidate drugs targeting the FDX1 and DLAT were acquired from DrugBank database (https://go.drugbank.com/). To validate the drug targets of FDX1 and DLAT, molecular docking was performed with AutoDock vina 1.1.2. The structures of FDX1 (PDB: 3P1M) and DLAT (PDB: 1Y8N) were obtained from the Research Collaboratory for Structural Bioinformatics (RCSB) Protein Databank (https://www.rcsb.org/). Their X-ray crystal structures: FDX1: Resolution: 2.54 Å, R-Value Free: 0.241, R-Value Work: 0.204, R-Value Observed: 0.205^21^; DLAT: Resolution: 2.60 Å, R-Value Free: 0.248, R-Value Work: 0.210, R-Value Observed: 0.211^22^. The results are analyzed and visualized by using PyMOL.

### Cell lines and clinical samples

Human ccRCC cell lines 786-O and A498 were purchased from the American Type Culture Collection (Rockville, MD, USA) and maintained at 37°C in a humidified, 5% CO_2_ controlled atmosphere in RPMI medium 1640 and MEM medium supplemented with 10% fetal bovine serum and 1% penicillin/streptomycin, respectively.

Tumor and adjacent non-tumorous tissues were taken from five patients with ccRCC who underwent surgical treatment in the First Hospital of Hunan University of Chinese Medicine. All procedures involving human participants in this study complied with the Declaration of Helsinki (2013 revision). This study was approved by the Ethics Committee of Hunan University of Chinese medicine (Ethics number: HN-LL-GZR-2022-22). Before surgery, all patients with ccRCC provided informed consent and received no further special therapy. When we conducted experiments using samples, patients provided oral informed consent due to age, cognitive ability, or other health conditions.

### CCK-8 assay

The CCK-8 assay was performed to explore elesclomol-induced cuproptosis in ccRCC. Cells at 5000 cells/well were seeded in 96-well plates and co-treated with different concentrations of elesclomol (0-100 nM) and 1µM CuCl_2_, incubated for 12 h at 37℃. Meanwhile, cells treated with elesclomol alone act as a control. CCK-8 solution (5 mg/mL) was added to each well at 10 µL. After 4 h of incubation. The absorbance of each well was detected by using an enzyme-labeled instrument (Bio-Rad Laboratories, Hercules, CA, USA) at a wavelength of 490 nm.

### Western blot assay

Total protein from cells and tumor tissue from ccRCC patients were extracted with RIPA lysis buffer containing proteinase inhibitor (Beyotime, Shanghai, China). Proteins were separated by SDS-polyacrylamide gel electrophoresis (SDS-PAGE) and treated with primary antibodies overnight at 4°C, which were washed and incubated with the peroxidase-conjugated anti-rabbit secondary antibody at room temperature for 1 h. Chemiluminescent detection was carried out utilizing ECL reagents with a ChemiDoc MP imaging system (Millipore Corporation). The following primary and secondary antibodies were used: antibodies for β-actin (1:2,000), FDX1 (1:2,000), DLAT (1:2,000) and peroxidase-conjugated anti-rabbit (1:5000). All antibodies were purchased from Abcam.

### Statistics

SPSS20.0 was used for statistical analysis of all data; and the results were presented as mean ± standard deviation (mean ± SD). The significant difference was analyzed by one-way analysis of variance or unpaired Student's t-test, and *P* < 0.05 was considered statistically significant. All data shown are representative of at least three independent experiments.

## Results

### Cuproptosis differential genes involved in ccRCC collection and functional enrichment analysis

**Figure 1:**
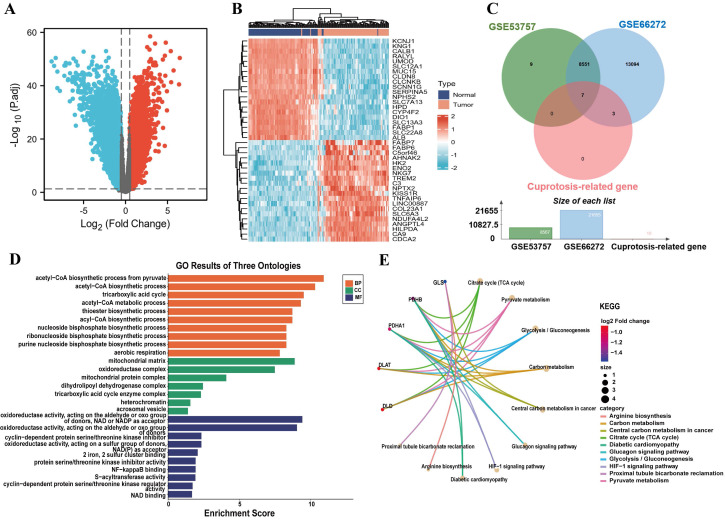
The identification of cuproptosis differential genes and enrichment analysis of function and pathway. (A) The volcano plot reveals differential genes in ccRCC. (B) Heatmap displays the top 20 significantly up- and down-regulated differentially expressed genes. (C) Venn diagram of cuproptosis differentially expressed genes. (D) GO functional enrichment analysis (BP, MF and CC). (E) KEGG pathway enrichment analysis of cuproptosis differential genes.

We downloaded the GSE53757 and GSE66272 microarray expression profiling datasets from the GEO database and obtained 8551 differential genes at |log2 (fold-change) | > 0.5 and *P* < 0.05 (Fig. 1A, 1C). In addition, cuproptosis-related genes were obtained including 10 genes from a previous study^15^ and intersected them with GSE53757 and GSE66272 to identify cuproptosis differential genes in ccRCC. The results indicated six down-regulated genes and only one up-regulated genes (Fig. 1C and Table S1). The Top 20 up- and down-regulated genes from ccRCC patients were exhibited in the heatmap (Fig. 1B). As we identified, functional and pathway enrichment analysis of seven cuproptosis differential genes was carried out using the online tool Metascape. The results of functional analysis revealed that acetyl-CoA biosynthetic process, acetyl-CoA metabolism process, acyl-CoA biosynthetic process, mitochondrial matrix, S-acyltransferase activity and Fe^2+^, 2 sulfur cluster binding were dramatically triggered in the gene sets (Fig. 1D). The activation of the citrate cycle (TCA cycle) signaling pathway, pyruvate metabolism, glycolysis/gluconeogenesis and carbon metabolism were all regulated by PDHB, PDHA1, DLAT and DLD; among them, PDHB and PDHA1 were involved in the glucagon signaling pathway, HIF-1 signaling pathway and diabetic cardiomyopathy (Fig. 1E).

### Validation and mapping of cuproptosis differential gene expression

**Figure 2:**
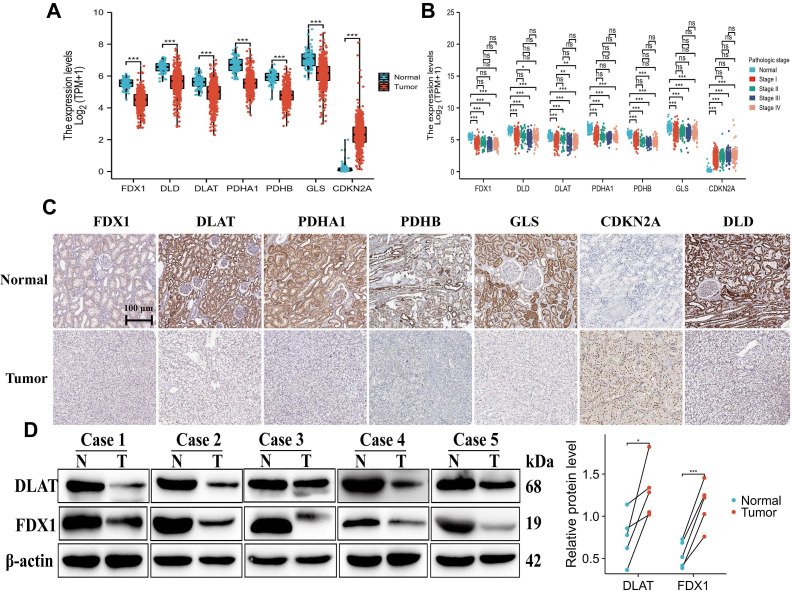
Expression and mapping analysis of cuproptosis differential genes. (A-C) The mRNA and protein expression patterns of cuproptosis differential genes in ccRCC by TCGA and HPA databases. (D) Western blot analysis of DLAT and FDX1 in five paired clinical ccRCC and paracancerous tissue samples. “ns” represents *P* ≥ 0.05, ^*^P < 0.05, ^**^P < 0.01, ^***^P < 0.001.

We further analyzed the mRNA expression of CDGs in ccRCC through the TCGA database (Unmatched renal clear cell carcinoma consisted of 72 paracancer tissues and 537 tumor samples). The results demonstrated that FDX1, DLD, DLAT, PDHA1, GLS and PDHB exhibited significantly lower expression in ccRCC tissues than normal tissues. Interestingly, only CDKN2A showed higher expression in ccRCC tissues than normal tissues (Fig. 2A). In addition, we found that the expressions of FDX1, DLD, DLAT, PDHA1, GLS and PDHB were significantly down-regulated in pathologic state, whereas CDKN2A was up-regulated as compared to comparable normal tissues (Fig. 2B). The results from the HPA database showed that protein expression levels of CDGs had also been consistent with mRNA expression in ccRCC (Fig. 2C). Based on the above results, we validated the protein expression of FDX1 and DLAT in five matched normal and ccRCC clinical samples, and found that their protein expressions were poorly expressed in ccRCC tissues compared to adjacent tissues (Fig. 2D). The results illustrated that cuproptosis differential genes were abnormally expressed in ccRCC patients in comparison to those of normal tissues, which might be predictive of poor prognosis and is related to disease stage progression in ccRCC patients.

### Effects of cuproptosis differential genes on ccRCC patient prognosis

**Figure 3:**
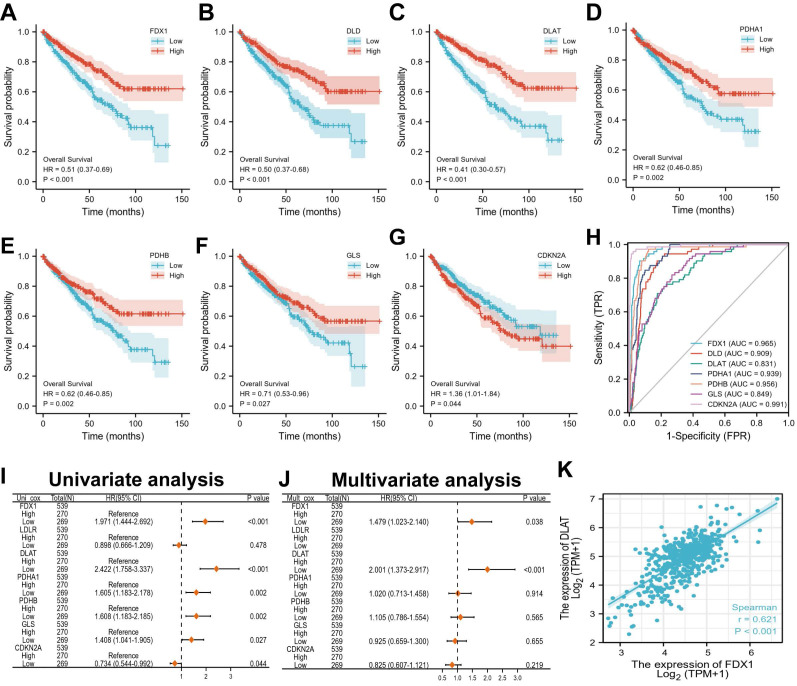
Overall survival and diagnostic value of different cuproptosis differential genes in ccRCC patients. (A-G) Prognostic analysis of cuproptosis differential gene expression in ccRCC patients. (H) The diagnostic value of different cuproptosis differential genes for ccRCC. (I, J) Univariate and multivariate regression model of differential genes for different cuproptosis. (K) Correlation analysis of FDX1 and DLAT genes.

Next, we evaluated the correlation between the expression of cuproptosis differential genes and the prognosis in ccRCC patients. The results indicated that ccRCC patients with low expression of FDX1, DLD, DLAT, PDHA1, GLS and PDHB had poor survival probability (HR < 1, *P* < 0.05), but not CDKN2A (Fig. 3A-G). Furthermore, the evaluation of univariate cox regression model revealed that low expression of FDX1 (HR 1.971; 95% CI 1.444-2.692, *P* < 0.001), DLAT (HR 2.422; 95% CI 1.758-3.337, *P* < 0.001), PDHA1 (HR 1.605; 95% CI 1.183-2.178, *P* = 0.002), PDHB (HR 1.608; 95% CI 1.183-2.185, *P* = 0.002) and GLS (HR 1.408; 95% CI 1.041-1.905, *P* = 0.027) were poor predictors for OS in ccRCC patients (Fig. 3I). Conversely, low CDKN2A expression (HR 0.734; 95% CI 0.544-0.992, *P* = 0.044) predicted a favorable prognosis in patients suffering from ccRCC. The multivariate cox results suggested that only FDX (HR 1.479; 95% CI 1.023-2.140, *P* = 0.038) and DLAT (HR 2.001; 95% CI 1.373-2.917, *P* < 0.001) were identified as independent prognostic factors affecting OS in patients with ccRCC (Fig. 3J). For the diagnostic value of cuproptosis differential genes in ccRCC patients, the AUCs of FDX1, DLD, DLAT, PDHA1, GLS, PDHB and CDKN2A were 0.965, 0.909, 0.813, 0.939, 0.956, 0.849 and 0.991, respectively (Fig. 3H). These findings suggest that ccRCC patients with strong (CDKN2A) or weak (FDX1, DLAT, etc.) CDGs expressions possess high diagnostic accuracy. Overall, FDX and DLAT act as independent prognostic factors and have a highly accurate diagnostic value for ccRCC patients. Thus, they may have important implications for the treatment of ccRCC patients. The correlation between FDX1 and DLAT in ccRCC was investigated by the “corrplot” R package. Notably, the results found that FDX1 and DLAT exhibited a strong positive correlation (r = 0.621, *P* < 0.01, Fig. 3K).

### Co-expression network selection and gene functional enrichment analysis of FDX1 and DLAT genes

**Figure 4:**
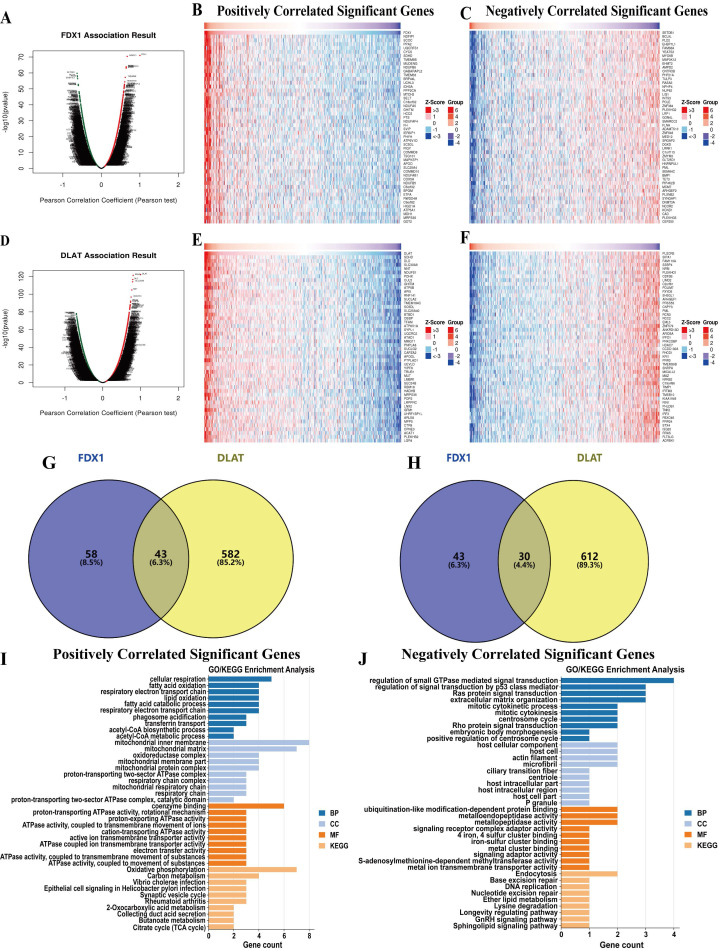
Differentially co-expressed genes that correlated with FDX1 or DLAT and functional analysis in ccRCC patients. (A) Correlations between FDX1 and differently expressed genes (Pearson correlation analysis). (B, C) Heatmaps show the genes positively or negatively associated with FDX1 (Top 50 genes). (D) Correlations between DLAT and differently expressed genes (Pearson correlation analysis). (E, F) Heatmaps show the genes that are positively or negatively correlated with DLAT (Top 50 genes). (G) The venn diagram results show that 43 genes are positively correlated with both FDX1 and DLAT. (H) The venn diagram results suggest that 30 genes are negatively correlation with both FDX1 and DLAT. (I, J) GO analysis and KEGG enrichment of the 43 and 30 genes, respectively.

We further identified differentially expressed genes related to FDX1 and DLAT in ccRCC using the LinkedOmics database. The differentially expressed genes associated with FDX1 and DLAT were found under the Pearson test (Fig. 4A, 4D), and their top 50 positively (r > 0) and negatively (r < 0) correlated genes were shown in the heatmap (Fig. 4B, 4C, 4E, 4F). The positive and negative correlation genes of FDX1 and DLAT were selected based on the following criteria: |r| > 0.5, *P* < 0.05. Eventually, 101 genes were positively and 83 genes were negatively correlated with FDX1 and 625 genes were positively and 642 were genes negatively correlated with DLAT, respectively. Among them, a total of 43 positively and 30 negatively associated genes with FDX1 and DLAT were identified for further analysis (Fig. 4G, 4H). These genes were used for GO and KEGG enrichment analysis. For genes positively associated with FDX1 and DLAT, the functional analysis demonstrated that acetyl-CoA biosynthetic process, respiratory electron transport chain, lipid oxidation, mitochondrial respiratory chain, active ion transmembrane transporter activity and acetyl-CoA metabolic process were severely affected. The KEGG results indicated that these genes were primarily involved in TCA cycle, oxidative phosphorylation and secretion, and carbon metabolism (Fig. 4I). For genes negatively correlated with FDX1 and DLAT, the functional analysis indicated that the regulation of signal transduction by p53 class mediator, mitotic cytokinetic process, host intracellular domains, iron-sulfur cluster binding, and metal cluster binding were impacted on the biological processes, molecular functions, and cellular component terms. The KEGG results revealed that these genes were mainly involved in the process of endocytosis, base excision repair and ether lipid metabolism (Fig. 4J).

### Genomic alterations and methylation analysis

**Figure 5:**
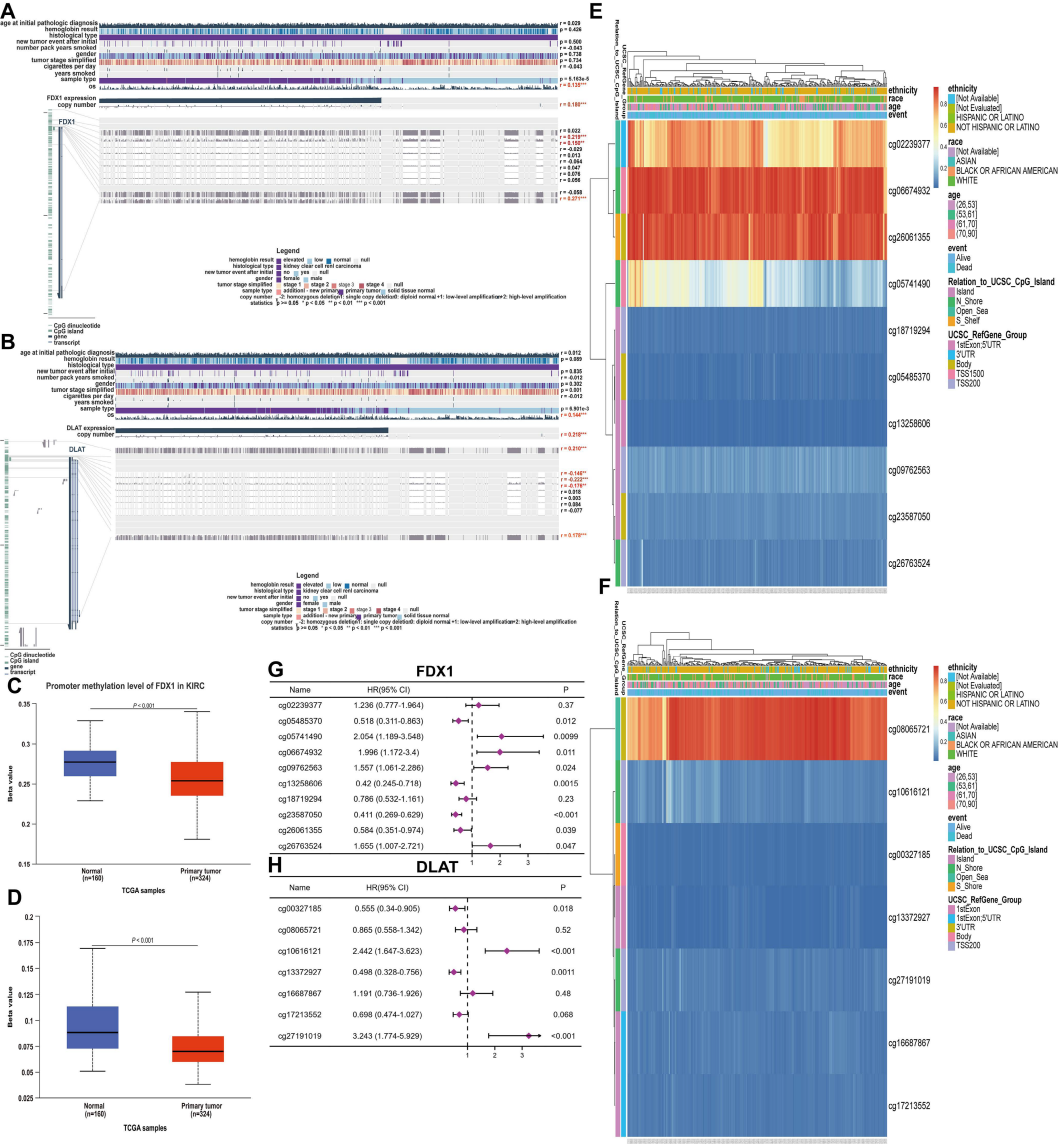
Methylation analysis of FDX1 and DLAT genes in ccRCC patients. (A, B) The methylation of FDX1 and DLAT was markedly correlated with OS and CpG islands in ccRCC patients. (C, D) The methylation levels of FDX1 and DLAT genes. The CpG methylation levels and forest plots of FDX1 (E, G) and DLAT (F, H).

The mutation frequencies of FDX1 and DLAT in ccRCC patients were explored through the cBioPortal database. The dataset including 392 patients (TCGA-ccRCC, Nature 2013, RNA Seq V2 RSEM) was selected for analysis. The somatic mutation frequency of DLAT in ccRCC was 0.3%, consisting mainly of missense mutations (Fig. S1B), which were comparatively rare, with only 1 in 392 samples. Surprisingly, FDX1 did not exhibit any somatic mutations (Fig. S1A). Therefore, FDX1 and DLAT mutations were not found to affect the overall survival of patients (Fig. S1C, S1D). Furthermore, we found that the total FDX1 and DLAT methylation levels were reduced in ccRCC tissues compared with normal tissues (Fig. 5C, 5D), and their methylation status was both dramatically correlated with OS and CpG sites in ccRCC patients (Fig. 5A, 5B). Thus, the methylation sites of FDX1 and DLAT genes were examined, along with the prognostic value of each CpG, based on the TCGA database. The data showed that cg02239377, cg06674932 and cg26061355 of FDX1 and cg08065721 of DLAT were the most methylated sites (Fig. 5E, 5F). Nevertheless, the methylation sites of the FDX1 gene included cg05485370 (HR:0.518, 95%CI: 0.311-0.863, *P* = 0.012), cg13258606 (HR:0.42, 95%CI: 0.245-0.718, *P* = 0.0015), cg23587050 (HR:0.411, 95%CI: 0.269-0.639, *P* < 0.001) and cg26061355 (HR:0.584, 95%CI: 0.351-0.974, *P* = 0.039), suggesting a good prognosis for patients suffering from ccRCC. In contrast, patients with cg05741490 (HR:2.054, 95%CI: 1.198-3.548, *P* = 0.0099), cg06674932 (HR:1.996, 95%CI: 1.172-3.4, *P* = 0.011), cg09762563 (HR:1.557, 95%CI: 1.061-2.286, *P* = 0.024) and cg26763524 (HR:1.655, 95%CI: 1.007-2.721, *P* = 0.047) conferred a poor prognosis (Fig. 5G). For DLAT gene, cg00327185 (HR:0.555, 95%CI: 0.34-0.905, *P* = 0.018) and cg13372927 (HR:0.498, 95%CI: 0.328-0.756, *P* = 0.0011) revealed a good prognosis in ccRCC patients, but cg10616121 (HR:2.442, 95%CI: 1.647-3.623, P < 0.001) and cg27191019 (HR:3.243, 95%CI: 1.774-5.929, P < 0.001) were associated with adverse patient outcomes (Fig. 5H).

### Correlation analysis of FDX1 and DLAT expression with immune infiltration level in ccRCC

**Figure 6:**
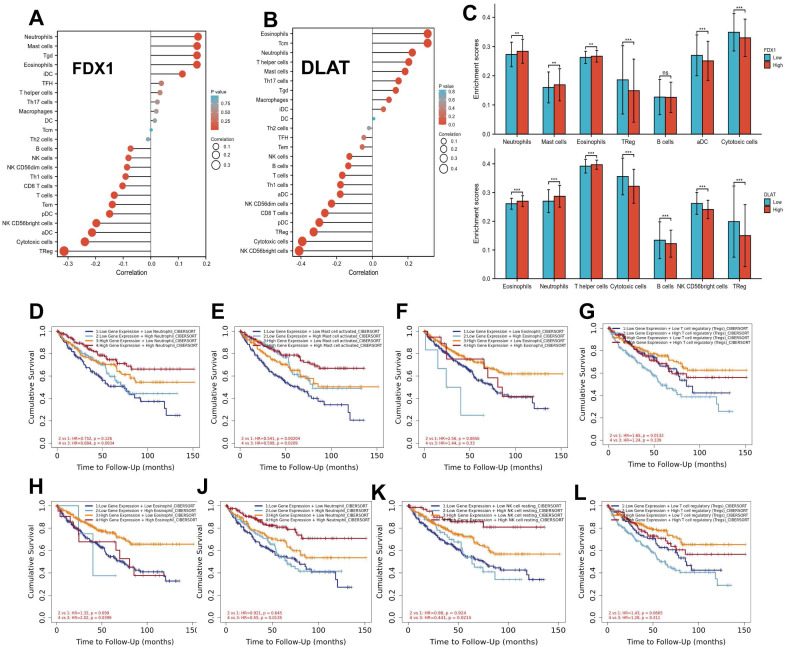
Correlation analysis of FDX1 and DLAT with the levels of immune cell infiltration. (A, B) The association of FDX1 and DLAT with 24 types of immune cells. (C) Comparison of immune cells between high- and low-FDX1 and DLAT expression groups. (D-G) Effects of neutrophils, mast cells, eosinophils and Treg levels on survival in ccRCC patients with high and low FDX1 expression. (H-L) Effects of eosinophils, neutrophils, NK cells and Treg levels on survival of ccRCC patients with high and low DLAT expression.

To understand the relationship between FDX1 and DLAT in cellular immunity, the potential correlation of their expression with 24 types of immune cells was analyzed by ssGSEA from the R package with a Spearman test. The findings showed that FDX1 expression was significantly related to neutrophils, mast cells, eosinophils, TReg, aDC and cytotoxic cells (Fig. 6A). However, DLAT was significantly associated with eosinophils, neutrophils, T helper cells, cytotoxic cells, B cells, NK CD56bright cells and TReg (Fig. 6B). Further studies demonstrated that FDX1 expression was positively correlated with infiltration levels of eosinophils and mast cells (r = 0.168, *P* < 0.001), neutrophils (r = 0.172, *P* < 0.001), but negatively correlated with cytotoxic cells (r = -0.239, *P* < 0.001) and TReg (r = -0.314, *P* < 0.001), while there was no significant correlation with B cells (r = -0.073, *P* < 0.090). DLAT expression was positively correlated with infiltration levels of eosinophils (r = 0.310, *P* < 0.001), neutrophils (r = 0.224, *P* < 0.001), T helper cells (r = 0.205, *P* < 0.001), but negatively correlated with CD8 T cells (r = -0.266, *P* < 0.001), cytotoxic cells (r = -0.393, *P* < 0.001) and B cells (r = -0.135, *P* < 0.002) (Fig. S2). Subsequently, the expression of FDX1 and DLAT were classified into high and low groups according to their expression levels. Significant differences were found in the levels of infiltrating immune cells, such as neutrophils, mast cells, eosinophils, Treg, DC and cytotoxic cells (*P* < 0.05), while FDX1 was not significant different in B cells. But for DLAT, there were obvious differences in the levels of infiltrating immune cells, including eosinophils, neutrophils, T helper cells, cytotoxic cells, B cells, NK CD56bright cells and TReg (*P* < 0.05) (Fig. 6C). Finally, we found that high levels of mast cells and Treg were remarkably associated with survival in ccRCC patients with low FDX1 expression (Fig. 6E, 6G), and high levels of eosinophils, neutrophils and NK cells were significantly correlated with survival in patients with high DLAT expression.

### Correlation analysis of FDX1 and DLAT expression with immune checkpoints

**Figure 7:**
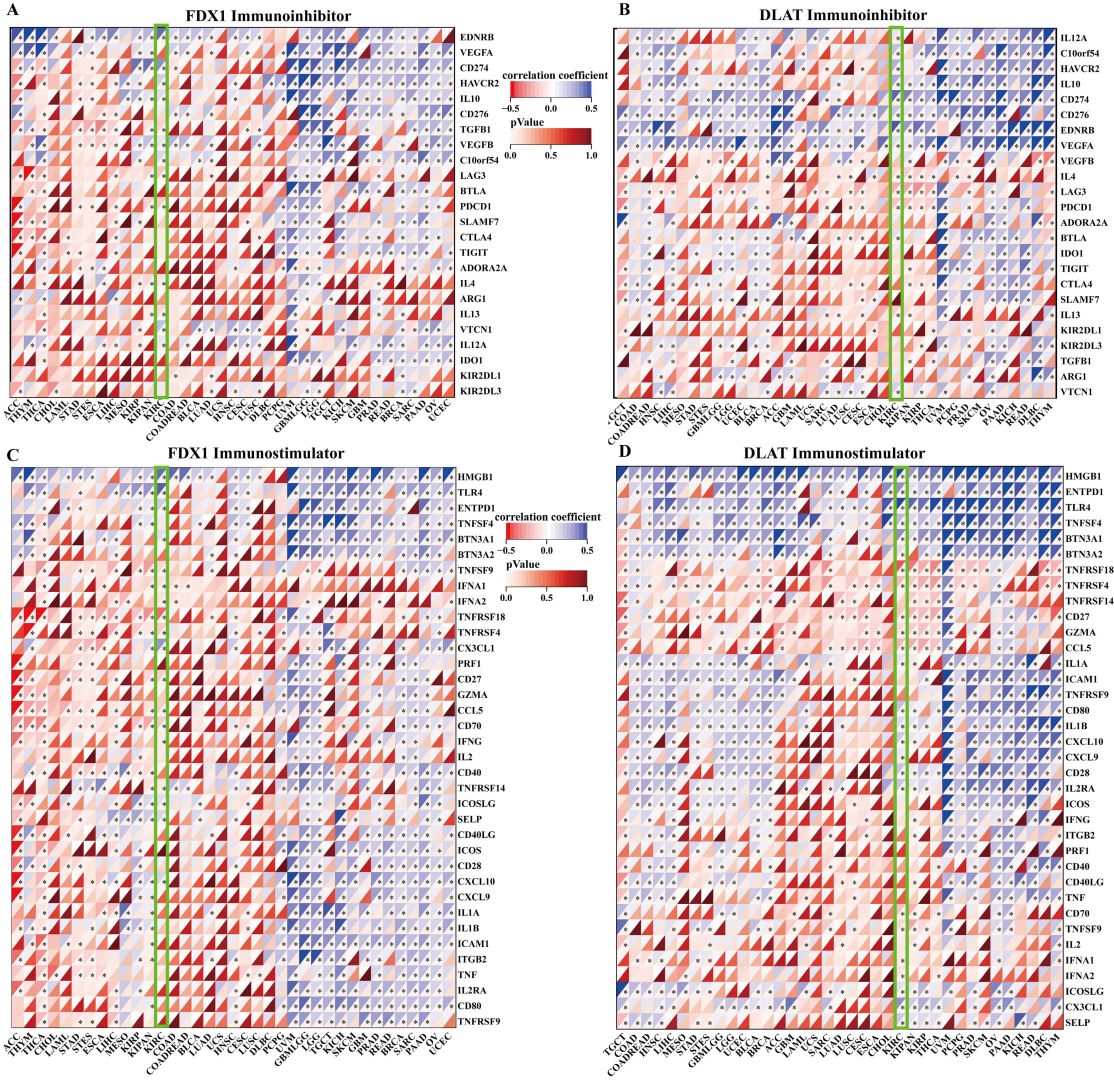
Correlation evaluation of immune checkpoint with the expression of FDX1 and DLAT. (A, B) The heatmaps displayed the correlation between immunoinhibitors with FDX1 and DLAT expression. (C, D) The heatmaps represented the correlation between immunostimulators with FDX1 and DLAT expression.

The correlation of FDX1 and DLAT genes with immunosuppressant checkpoints in pan-cancer were shown in Fig. 7A, 7B. Specially, FDX1 and DLAT genes were closely associated with EDNRB, CD274, HAVCR2, IL10, TGFB1, LAG3, PDCD1, IL4 and VTCN1 in ccRCC patients. Further studies revealed that FDX1 was positively correlated with CD274 (r = 0.309, *P* <0.001) and EDNRB (r = 0.421, *P* <0.001) (Fig. S3A, B) according to the following criteria: |r| > 0.3 and *P* <0.05. DLAT was also related to CD274 (r = 0.415, *P* <0.001) and EDNRB (r = 0.418, *P* <0.001) (Fig. S3C, D). Additionally, the correlations between immunostimulants and FDX1 and DLAT genes was investigated by Spearman correlation. The results showed that FDX1 and DLAT expression were markedly correlated with HMGB1, TLR4, CX3CL1, CD27, CCL5, CXCL10 and TNFRSF18 (Fig. 7C, 7D). We found that FDX1 expression was positively correlated with CX3CL1 (r = 0.395, *P* <0.001), TLR4 (r = 0.353, *P* < 0.001) and HMGB1 (r = 0.466, *P* <0.001), but negatively correlated with TNFRSP18 (r = -0.342, *P* <0.001) (Fig. S3E-H). As of interest, DLAT expression was also positively correlated with CX3CL1 (r = 0.331, *P* <0.001), TLR4 (r = 0.551, *P* < 0.001) and HMGB1 (r = 0.448, *P* <0.001), but negatively correlated with TNFRSP18 (r = -0.461, *P* <0.001) in patients with ccRCC (Fig. S3I-L). These results suggest that FDX1 and DLAT genes are highly relevant to immune checkpoints, which may lead to a favorable response from patients to immune checkpoint therapy.

### Exploring the sensitivity of ccRCC to cuproptosis

**Figure 8:**
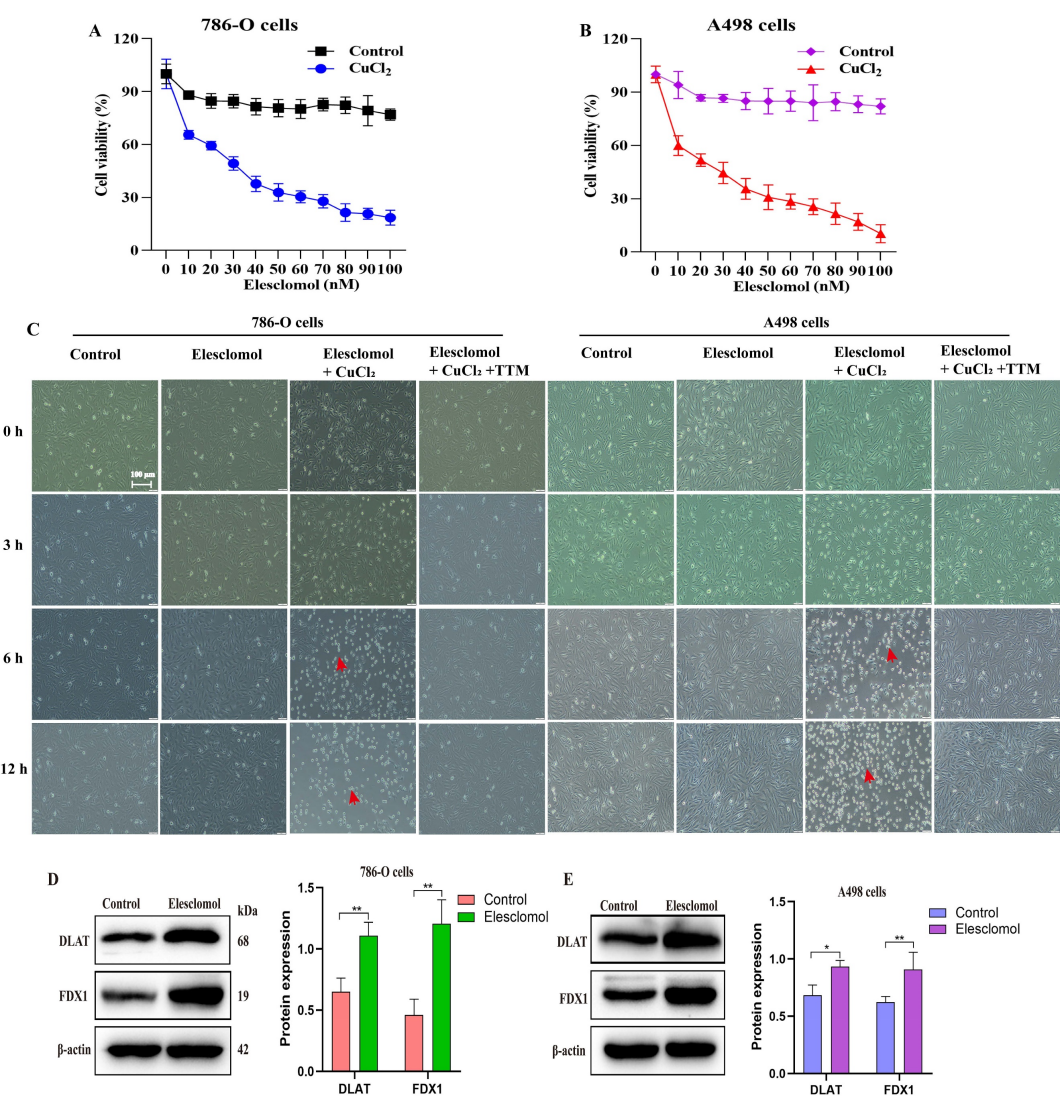
FDX1 and DLAT are pivotal targets of elesclomol-induced cuproptosis in ccRCC. (A, B) The cell viability of 786-O and A498 cells treated with elesclomol or elesclomol with CuCl_2_ (1 µM) for 12 h. (C) Representative brightfield images of 786-O and A498 cells incubated with either vehicle, 1µM CuCl_2_ or 1µM CuCl_2_ with 20 µM tetra-thiomolybdate (TTM), and then treated with 100 nM of elesclomol for 3, 6 and 12 h. (D, E) The expression of FDX1 and DLAT proteins in 786-O and A498 cells co-treated with elesclomol (30 nM) and CuCl_2_ (1 µM) for 12 h. Scale bar: 100 μm. ^*^P < 0.05, ^**^P < 0.01,^ ***^p < 0.001 *vs*. normal or control. All experiments were repeated independently three times.

To explore the sensitivity of ccRCC to cuproptosis, we analyzed the cytotoxic effect of the cuproptosis inducer elesclomol in the presence of CuCl_2_ on 786-O and A498 cells. The results profiled that different elesclomol (0-100 nM) induced cell death in a concentration-dependent manner. Contrarily, cells grown in the absence of CuCl_2_ were resistant to elesclomol (Fig. 8A, 8B). The brightfield images of cell growth showed that co-treatment with elesclomol (100 nM) and CuCl_2_ (1 µM) resulted in the death of 786-O and A498 cells after 6 and 12 hours, while chelating copper with 20 µM tetra-thiomolybdate (TTM) prevented cell death (Fig. 8C). Of note, the expression of both FDX1 and DLAT proteins was up-regulated when 786-O and A498 cells were co-treated with 30 nM elesclomol and 1 µM CuCl_2_ (Fig. 8D, 8E).

### Screening and validation of potential drug targets for FDX1 and DLAT

**Figure 9:**
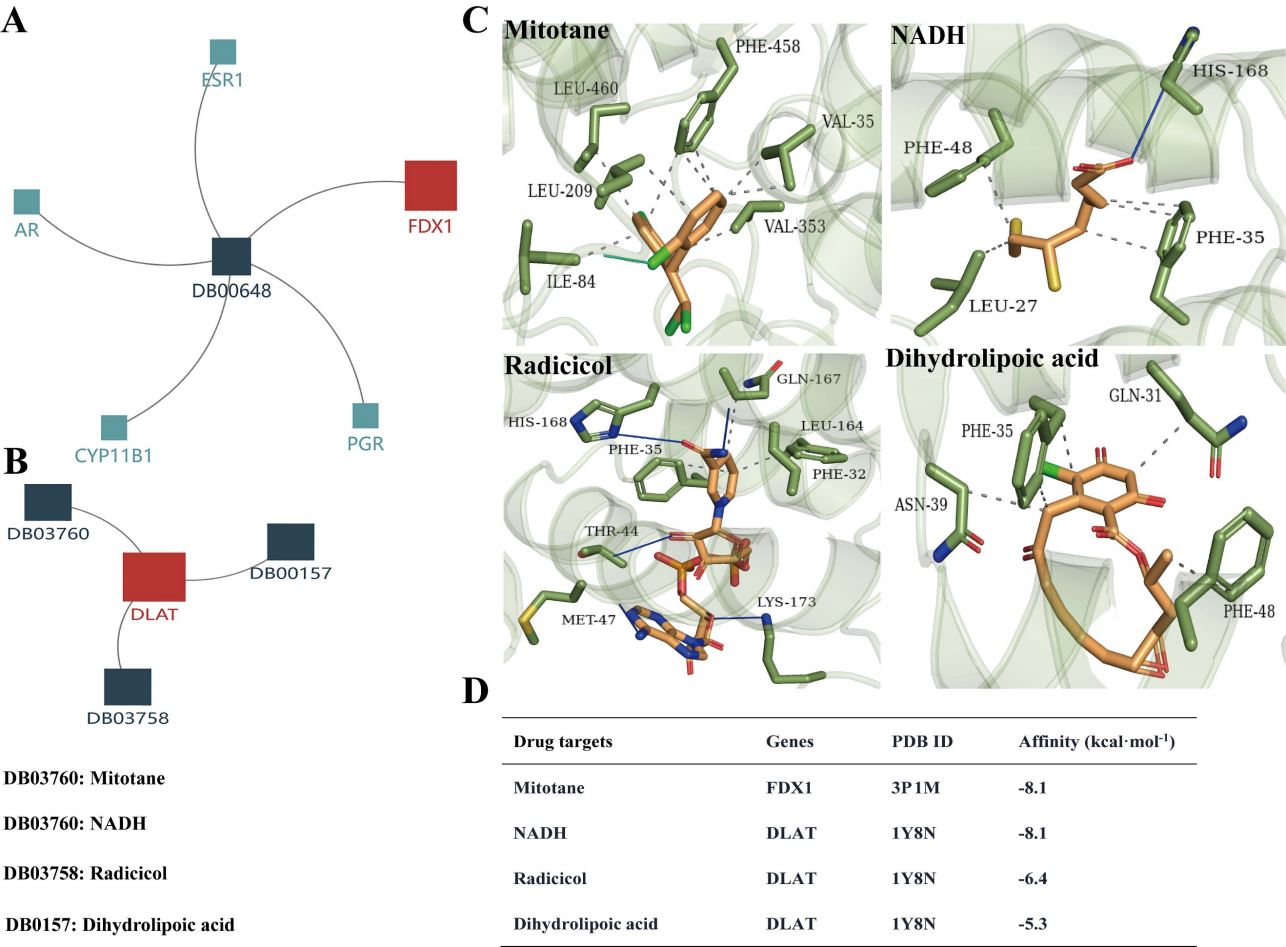
The drug targets of FDX1 and DLAT proteins. (A, B) Illustrations represent the drug targets of FDX1 and DLAT. (C) Three-dimensional illustrations displayed interactions of mitotane, NADH, adicicol and dihydrolipoic acid in the amino acid sites of FDX1 and DLAT proteins. (D) The affinity and related information of drug targets for FDX1 and DLAT. Blue solid line: hydrogen bonds; Grey dotted line: hydrophobic interaction. NADH: beta-Nicotinamide adenine dinucleotide.

Based on the above analysis results showing that FDX1 and DLAT are promising targets for ccRCC therapy, we predicted the corresponding drug targets through the DrugBank database, a drug target information source. The data indicated that FDX1 was assigned only one agent named mitotane, whereas NADH, radicicol and dihydrolipoic acid are potential drug targets for DLAT (Fig. 9A, 9B). Molecular docking results revealed that Leu460/209, Phe458, Val35/353 and Ile84 of FDX1 were key sites for mitotane binding, its binding affinity up to -8.1 kcal. mol^-1^. NADH, radicicol and dihydrolipoic acid docked to several critical sites of DLAT, including Phe48/35/32, His168, Met47, Asn39, Gln31, etc., and the binding affinities were -8.1 kcal. mol^-1^, -6,4 kcal. mol^-1^ and -5,3 kcal. mol^-1^, respectively (Fig. 9C, 9D).

## Discussion

Copper is one of the most important trace elements in the human body, integral to various essential proteins, and involved in extremely relevant physiological and biochemical processes^23^, ^24^. The metabolic abnormalities of copper can cause related diseases, such as immune dysfunction, anaemia and endocrine disorders^25^. Intracellular copper overload leads to cell death, termed cuproptosis, and is considered a state-of-the-art strategy for the treatment of cancers^15^. However, few reports on the therapeutic strategies and mechanisms behind cuproptosis in a variety of tumors. Therefore, extensive works are required to further investigate the underlying mechanism and potential targets.

In the present study, we found that ccRCC was highly sensitive to cuproptosis and identified cuproptosis-related genes FDX1 and DLAT as the most prognostic and diagnostic candidates for ccRCC. FDX1, one of the ferredoxin families, contains soluble iron-sulfur (FeS) proteins^26^. Importantly, FDX1 encodes FeS protein that reduces Cu^2+^ to Cu^1+^ and is engaged in protein lipoylation in the TCA cycle, thereby regulating cuproptosis^15^, ^27^. DLAT is a mitochondrial protein that is an integral component of the pyruvate dehydrogenase complex (PDH)^28^ and oligomerizes because the integration of copper and lipid acylated proteins in the TCA cycle plays an important role in cuproptosis^15^. Noteworthy, we found that the expressions of FDX1 and DLAT was lower in patients with ccRCC than in normal tissues. The high expression of both FDX1 and DLAT underscores the great clinical significance and diagnostic value, correlating with the favorable survival in ccRCC patients. These results highlight that they may serve as possible therapeutic targets against ccRCC. Emphatically, the relationship between FDX1 and DLAT exhibited a strong positive correlation. Previous evidence indicated that FDX1 leads to the accumulation of the toxic lipoylated DLAT, resulting cuproptosis^15^, ^29^. Protein lipoylation is a highly conserved post-translational modification of lysine observed on only four enzymes: dihydrolipoamide branched-chain transacylase E2 (DBT), glycine cleavage system protein H (GCSH), dihydrolipoamide S-succinyltransferase (DLST), and these enzymes participate in regulating the TCA cycle^30^, ^31^. The lipoylation of these proteins is essential for the proper function of these enzymes. Furthermore, FDX1 is an upstream regulator of protein lipoylation. This might explain the positive correlation between FDX1 and DLAT in ccRCC. Next, focusing on the functional analysis of the differentially expressed genes that are positively and negatively associated with both FDX1 and DLAT genes in ccRCC. It is revealed that these genes were mainly involved in the process of acetyl-CoA metabolism, acetyl-CoA biosynthesis, lipid oxidation, mitochondrial respiratory chain, acetyl-CoA metabolism and iron-sulfur cluster binding.

Gene mutations across tumor progression and treatment failure, such as drug resistance and immunotherapy resistance. The common tumor suppressor gene TP53 is frequently deleted or mutated in human cancers and is involved in the progression of drug resistance in carcinomas^32^, ^33^. Mutations in JAK1, JAK2 and STAT1 gene induce resistance to immunotherapy in patients with metastatic melanoma^34^. Most studies focus on the survival prognosis, clinical features, methylation, and biological functions of the cuprotosis-related gene FDX. However, there is little research on the significance of DLAT methylation and mutation in cuprotosis in ccRCC. Accumulating evidence suggests that FDX1 mutations result in permanent neonatal diabetes with subclinical exocrine deficiency^35^, while DLAT mutations cause atypical Pantothenate Kinase Associated Neurodegeneration (PKAN)^36^. Our data indicated that the likelihood of mutations in FDX1 (with no mutations observed) and DLAT (0.3%) is very low in ccRCC. This reason could be that many important genes are not mutated or deleted, and aberrant gene expression is achieved through DNA methylation in cancer^37^, ^38^. The correlation between methylation and gene expression levels is complex and can be positive or negative, and the causality of this relationship remains unclear^39^. Our results revealed that the promoter methylation levels of FDX1 and DLAT were substantially reduced in ccRCC compared to normal tissues, suggesting that the changes in FDX1 and DLAT expression are due to this epigenetic modification. A natural follow-up issue is whether methylation of FDX1 and DLAT can be useful as prognostic predictors in patients with ccRCC. Earlier work has reported that the hereditary genetic polymorphisms of ATG5 serves as a prognostic predictor in patients with esophageal squamous cell carcinoma^40^. The study indicated the methylated CpG sites of FDX1 were beneficial for OS, and DLAT gene has only two methylated CpG sites beneficial to OS. The relationship between DNA methylation and gene expression largely depends on the genomic location of DNA methylation, which also influences the clinicopathological features of cancers^41^, ^42^. This may explain the different prognostic significance of FDX1 and DLAT methylation sites in ccRCC patients.

ccRCC is an immunogenic cancer typically infiltrated by immune cells, including macrophages and T lymphocytes^43^. In particular, T cells are the most abundant immune infiltrating cells in ccRCC^44^. Higher levels of tumor-infiltrating lymphocytes and CD8^+^T cells have been proven to be associated with poorer survival in ccRCC patients^45^-^47^. In this study, we found that FDX1 and DLAT were inversely correlated with the infiltration of most immune cells, particularly Tregs and CD8^+^ T cells. The expressions of FDX1 and DLAT are tightly associated with immune checkpoints, including PD-1, LAG3, PD-L1 and TLR4. Previous studies consistently reported that the expressions of LAG-3, PD-1, PD-L1, CD28, CD80, CD27 and CTLA-4 were relevant to the immunosuppression in the tumor microenvironment^48^-^50^. LAG3, CD28, CTLA4, PD-L1 have been identified as adverse prognostic factors in ccRCC patients^48^, ^51^. PD-1 is highly expressed in ccRCC tissues, which increases the infiltration of CD8^+^ T cells and Tregs, leading to immunosuppression within the immune microenvironment^52^, ^53^. Consistent with the above literature, our study explains that high levels of immune infiltration checkpoints lead to suppression of the tumor immune microenvironment. However, FDX1 and DLAT genes are highly correlated with immune checkpoints, which result in favorable patient responses to immune checkpoint therapy. More importantly, we confirmed the low expression of FDX1 and DLAT in clinical ccRCC samples and the sensitivity of ccRCC to cuproptosis* in vitro*. Furthermore, we analyzed the cytotoxic effects of cuproptosis inducer elesclomol in the presence of CuCl2 on ccRCC cells (786-O and A498) in the present study. The results showed that elesclomol reduced the cell viability in a concentration-dependent manner, whereas Cu chelator TTM prevented cell death of ccRCC. FDX1 as a potential target for mitotane and DLAT as a potential target for NADH, radiaticol, and dihydrolipoic acid were predicted by DrugBank database. Existing literatures have reported the antitumor activity of mitotane, adicicol and dihydrolipoic acid^54^-^56^, but its molecular mechanisms still remains to be elucidated. The molecular docking results indicated that FDX1 and DLAT might be potential drug targets for mitotane, adicicol and dihydrolipoic acid against ccRCC by inducing cuproptosis. Despite these intriguing results, our study inevitably has some shortcomings. These results need to be further validated by biological experiments.

## Conclusion

In the present study, we investigated the sensitivity of ccRCC to cuproptosis and applied comprehensive bioinformatics analysis to reveal the roles of cuproptosis-related genes FDX1 and DLAT in ccRCC. The findings demonstrated that both FDX1 and DALT were independent prognostic markers for poor survival in ccRCC patients, showing a strong positive correlation as well as the fact that they were correlated significantly with immune infiltration and immune checkpoints. Taken together, FDX1 and DLAT are promising targets for the prevention of ccRCC, and cuproptosis may be a novel and attractive strategy for therapy of ccRCC. The combination of targeting FDX1 and DLAT may be a novel insight into the induction of cuproptosis in ccRCC.

## Supplementary Material

Supplementary figures and table.

## Abbreviations

ccRCC: clear cell renal cell carcinoma
CDGs: cuproptosis differential genes
OS: overall survival
PCD: programmed cell death
TCA: tricarboxylic acid
ROC: receiver operating characteristic
GO: gene ontology
KEGG: kyoto Encyclopedia of Genes and Genomes
FDX1: ferredoxin 1
DLAT: dihydrolipoyl transacetylase
ssGSEA: single-sample GSEA
TIMER: Tumor Immune Estimation Resource
SDS-PAGE: SDS-polyacrylamide gel electrophoresis
TCA cycle: the citrate cycle
FeS: iron-sulfur
PDH: pyruvate dehydrogenase complex
DBT: branched-chain transacylase E2
GCSH: glycine cleavage system protein H
DLST: dihydrolipoamide S-succinyltransferase
