# Supplementary material for: Sensitivity of renal cell carcinoma to cuproptosis: a bioinformatics analysis and experimental verification
Source: J Cancer. 2025 Jan 1;16(3):952–68. doi: 10.7150/jca.103708 (PMC11705067; doi:10.7150/jca.103708)
Supplement: Supplementary file 1 — Supplementary figures and table. [file jcav16p0952s1.pdf]

Supplementary Material

Sensitivity of renal cell carcinoma to cuproptosis: A bioinformatics analysis and experimental verification

Hongfang Li[1], Chanjuan Zhang[1], Neng Zhu[2], Yaning Shi[1], Li Qin[1,3,4,✉]

[1] Laboratory of Stem Cell Regulation with Chinese Medicine and Its Application, School of Pharmacy, Hunan University of Chinese Medicine, Changsha 410208, China

[2] Department of Urology, The First Hospital of Hunan University of Chinese Medicine, Changsha 410007, China

[3] Institutional Key Laboratory of Vascular Biology and Translational Medicine in Hunan Province, Changsha, China

[4] Hunan Province Engineering Research Center of Bioactive Substance Discovery of Traditional Chinese Medicine, Hunan University of Chinese Medicine, Changsha, China.

✉ Corresponding author: Li Qin, E-mail: lqin@hnucm.edu.cn; ORCID: 0000-0002-9797-2636

Supplementary tables:

Table S1 Cuproptosis differentially expressed genes of ccRCC in the GES53757 dataset

Genes (Human)	Gene title	Fold change	P value	Regulation direction
**FDX1**	Ferredoxin-1	-1.3653	2.41375E-41	down
**DLD**	Dihydrolipoamide dehydrogenase	-0.8781	8.78061E-27	down
**DLAT**	Dihydrolipoamide S-acetyltransferase	-0.8640	6.06852E-29	down
**PDHA1**	Pyruvate dehydrogenase E1 subunit alpha 1	-1.1229	4.4676E-40	down
**PDHB**	Pyruvate dehydrogenase E1 subunit beta	-1.2590	2.39053E-46	down
**GLS**	Glutaminase kidney isoform	-1.5625	6.40321E-35	down
**CDKN2A**	Cyclin-dependent kinase inhibitor 2A	1.3286	4.17763E-30	up

Supplementary figures

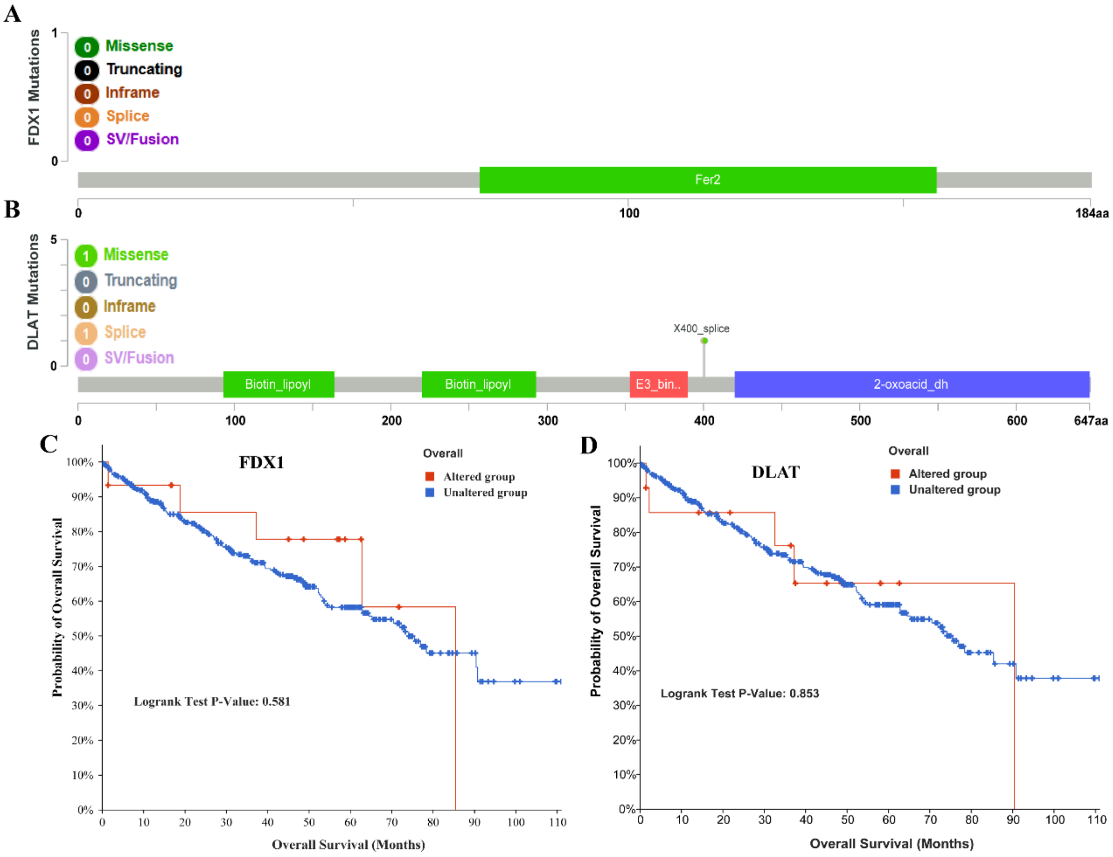

Figure S1: FDX1 and DLAT mutations in ccRCC patients. (A, B) Illustration of FDX1 and DLAT gene mutations in ccRCC by the online cBioPortal tool. (C, D) Overall survival with alterations in FDX1 and DLAT in ccRCC patients.

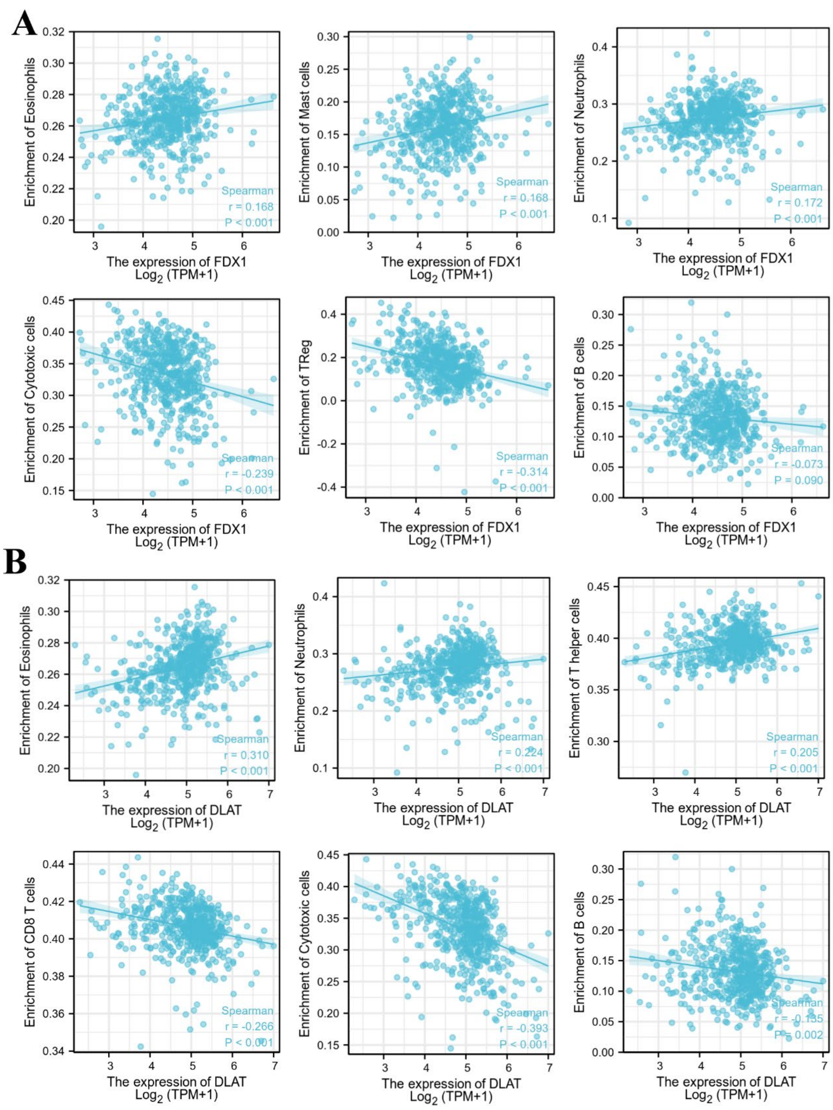

Figure S2: Correlation of FDX1 and DLAT expression with levels of immune cell infiltration. (A) The expression of FDX1 gene was associated with eosinophils, mast cells, neutrophils, cytotoxic cells, Treg and B cells. (B) DLAT expression was related to eosinophils, neutrophils, T helper cells, CD8 T cells, cytotoxic cells and B cells.

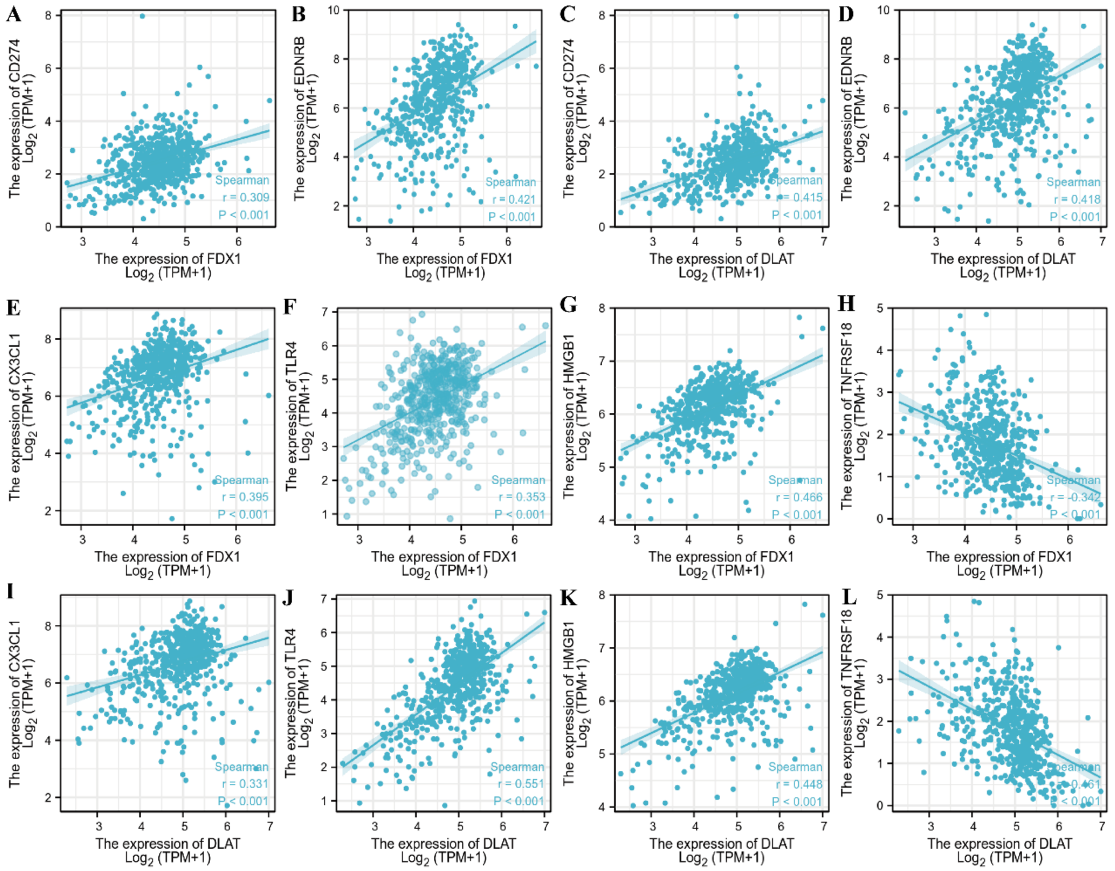

Figure S3: Correlation evaluation of immune checkpoints with the expression of FDX1 and DLAT. (A-D) Both FDX1 and DLAT expressions in ccRCC were positively correlated with the immunosuppressants CD274, and EDNRB. (E-L) FDX1 and DLAT expression in ccRCC were all significantly associated with the immunostimulants CX3CL1, TLR4, HMGB1 and TNFRSF18.
